# Variations in limited resources allocation towards friends and strangers in children and adolescents from seven economically and culturally diverse societies

**DOI:** 10.1038/s41598-022-19354-7

**Published:** 2022-09-08

**Authors:** M. Butovskaya, V. Rostovtseva, D. Dronova, V. Burkova, Y. Adam

**Affiliations:** 1 Institute of Ethnology and Anthropology of the Russian Academy of Sciences, Leninskiy pr-t, 32a, Moscow, Russia 199334; 2 National Research University Higher School of Economics, Moscow, Russia; 3 Russian State University for the Humanities, Moscow, Russia

**Keywords:** Anthropology, Behavioural ecology, Cooperation

## Abstract

Humans are unique among primates in altruism and sharing limited recourses towards non-kin. Our study revealed the differences in proportions of individuals ready to share limited resources with virtual friend compared to virtual stranger in children and adolescents from seven ethnic groups, represented by four traditional rural African societies from Tanzania with different types of economy and three societies from Russia. The study was conducted between 2015 and 2020, and the data on 2253 individuals (1104 males and 1149 females) were obtained. Six economic games with limited resource allocations were conducted: Prosocial, Envy, and Sharing games with imagined friends and stranger partners accordingly. All players were later classified according to their decisions in all six games into four behavioral types: egoistic, egalitarian, altruistic, and mixed. The effects of population origin, gender, age, and stranger/friend type of interaction on the behavior were estimated by multinomial logistic regression. It was demonstrated that more respondents prefer altruistic and egalitarian behavior than egoistic and mixed in the whole sample. However, significant parochial effect was found. The study revealed significant main effects of ethnicity, age, and the interaction effects of ethnicity and parochial tendencies, and ethnicity and age on the behavior of players.

## Introduction

Humans are unique among primates for the degree of altruism and sharing towards unrelated individuals, including strangers^1–4^, although few cases of out-group sharing have been recently registered in bonobo^5^. As a rule, universally adults are highly motivated to develop a reputation of cooperative partner^3^, and reputation for being generous is of key-importance for gaining social status and prestige in many traditional societies^6^.

Along with group-level selection for altruism and sharing, humans were positively selected for being generous and empathetic towards familiar others^7^. Predisposition to altruism could be fixed at the genetic level, hence there is a possibility that individual sharing strategies may vary due to this factor, along with socialization experience, group social norms, as well as ecological environment (food availability and demands for its acquisition).

Social cooperation is a corner stone of human society and food-sharing as one of the forms of cooperation may be seen as unique human evolutionary adaptation for emergence of durable social networks between unrelated individuals. Food-sharing has been driven by a number of factors, environmental, economic, and political in the first rate^8^. The size and ramification of food sharing and cooperation networks vary greatly according to ethnographic data^9^, and these variations may be due to economy type and typical diet^10–14^. Food-sharing norms may accentuate reciprocation. This is especially important for survival in immediate-return societies, where food is not stored, and people are highly susceptible to hunger threat in the case of individual failure in food acquisition^15^. Hadza children were reported to be highly reciprocal in this respect^16^. Children from rural societies with more group-oriented values distribute resources more fairly compared to their peers from more individualistic societies^17^. In delayed-return societies, individuals can protect themselves from famine through food production and storage^18^, and frequent food-sharing may not be that crucial for survival. In modern postindustrial societies these tendencies are even more obviously present. Hence, we may expect sharing limited recourses in this case will drop substantially in frequency, and these may be evident not only among adults, but among children as well.

According to a number of studies, with age children and adolescents become more parochial, and share more with in-group versus out-group peers^19^. With age children also become more strategic and driven by consideration of efficiency when taking sharing decisions^20,21^, and less spiteful^19^. Already 3-year-old children give more resources to their friends than to strangers^22^, and 3–5-year-old children distribute more resources to members of their own gender and race^23^.

Recent data on sharing behavior across 12 countries^24^ suggest that children may learn the principles of sharing simply by getting information about other’s behavior. Samek with colleagues^24^ conducted a dictator game, in which children could share up to 10 of their stickers with another anonymous child. Children were randomly divided into two groups. One of which was assigned “shared a little” and the other “shared a lot” option. In the first case children were told that another child had shared 1 sticker, whereas in the second case that another child had shared 6 stickers in the same game. The “shared a lot” option revealed the overall positive effect on sharing. Hence, according to Samek and coauthors^24^, the social information about sharing by other peers is important for children’s decision making. No differences between collectivist and individualist cultures were found in this study. Hence, the collectivist-individualist dimension itself may not be the primary factor explaining the effect of social information on children’s decision making. To what extent the members of individualistic and collectivistic societies differ in decisions on limited resources allocation remained to be investigated more thoroughly.

Several cross-cultural studies elucidated obvious differences in helping and sharing in children and adults^9,17,24–29^. However, to what extent children and adolescents of different age and gender from non-Western, traditional cultures may differ from Western peers in their sharing strategies towards in-group and out-group members remained to be investigated more carefully. These considerations determined the goals of our study: to estimate the differences in the proportions of children and adolescents practicing egoistic, egalitarian, altruistic, and mixed strategies in sharing limited resources towards friends and strangers accordingly, and to reveal the role of age, gender and cultural origin in differences obtained. Seven ethnic groups, represented by four small-scale rural African societies from Tanzania and three societies from Russia (both rural and urban) were tested in this study. African societies were represented by Hadza (nomadic hunter-gatherers), Iraqw (agro-pastoralist), Meru (intensive agriculturalists), and Haya (intensive agriculturalists). Societies from Russia included Buryats (traditionally nomadic pastoralists of Mongolian origin from Southern Siberia; both rural and modern urban groups were tested), Tuvans (descendants of pastoralists and reindeer herders from Southern Siberia; rural group was studied), and Russians (modern industrial society; both urban and rural groups were studied). In total, the data on 2253 individuals of both sexes and between 5 and 20 years of age were collected.

The prosocial orientations of children and adolescents were evaluated applying an experimental method, originally developed by Fehr with co-authors^30^. Every child played three types of games (Prosocial, Envy, and Sharing games), where had to make decisions about allocation units of candies to himself/herself and/or to an imagined partner who was either a friend or a stranger (both options were tested for each participant). In the Prosocial game basic predispositions for prosociality were tested (giving or not giving a candy to a partner at no cost to oneself). In the Envy game propensity for jealousy was tested (giving one or two candies to a partner at no cost to oneself). In the Sharing game basic predisposition for altruism was tested (sharing or not sharing a candy with a partner with a real cost to oneself). In the course of the experiment, participants from all studied populations suggested deviant decisions (5.7%), which corresponded to extremely selfish or extremely prosocial individual choices. These decisions were kept in the analysis (for details see Methods). Thus, our approach has been considerably modified from the original one^19,30^, and shifted the emphasis from the behavioral economics to the ethological perspective. The summary of all considered decisions in all three games is presented in Table 1.

**Table 1:** Experimental games.

Games		Allocation decision [option 1]	Allocation decision [option 2]	Allocation decision [option 3]
1	Prosocial game	1 _SELF_ : 0 _PARTNER_	1_SELF_ : 1 _PARTNER_	
2	Envy game	1 _SELF_ : 0 _PARTNER_*	1 _SELF_ : 1 _PARTNER_	1 _SELF_ : 2 _PARTNER_
3	Sharing game	2 _SELF_ : 0 _PARTNER_	1 _SELF_ : 1 _PARTNER_	0 _SELF_ : 2 _PARTNER_*

Three games: in each game a player had to make a decision of how to allocate candies between himself/herself and/or a virtual partner. Each game was played twice: once toward an anonymous friend, and once toward an anonymous stranger of the same age.

* decisions that deviate from the original method.

Subsequently, all subjects were classified according to their decisions in all three games (towards friends or strangers accordingly) into four behavioral types: egoistic, egalitarian, altruistic, and mixed strategy followers. Egoistic individuals were striving to minimize their partner’s payoffs; egalitarians had a propensity for inequality aversion in that they tended to reach equal allocations of candies between self and a partner; altruists were those who made decisions that maximized their partner’s payoffs independently of the own cost; mixed category included subjects whose behavior did not fit into any of the three previously mentioned types. The summary of classification into behavioral types is presented in Table 2.

**Table 2:** Classification of Behavioral Types.

Behavioral types		Prosocial game allocation	Envy game allocation	Sharing game allocation
1	Egoistic	1 _SELF_ : 0 _PARTNER_	1 _SELF_ : 0 _PARTNER_*; 1_SELF_ : 1 _PARTNER_	2 _SELF_ : 0 _PARTNER_
2	Egalitarian	1_SELF_ : 1 _PARTNER_	1_SELF_ : 1 _PARTNER_	1_SELF_ : 1 _PARTNER_; 2 _SELF_ : 0 _PARTNER_
3	Altruistic	1_SELF_ : 1 _PARTNER_	1_SELF_ : 2 _PARTNER_	1_SELF_ : 1 _PARTNER_; 0 _SELF_ : 2 _PARTNER_*
4	Mixed	All other combinations		

Combinations of decisions that formed each behavioral type.

* decisions that deviate from the original method.

Summarizing the data from the literature reviewed above, we hypothesized the following. 1. In all investigated groups four types of individuals will be present: egoists, egalitarianists, altruists, and mixed-strategy followers. This hypothesis is based on the results of similar experiments in other societies, and is aimed to test for relative universality of human behavior. 2. In all groups the proportion of altruists and egalitarianists will be higher in case of sharing with friends compared to sharing with strangers. This result would demonstrate the universal nature of human parochialism, developed in the long course of human evolution. 3. In the samples from traditional African rural societies the proportion of altruists and egalitarianists will be higher both in the case of friends and strangers, compared to samples from industrial societies. This hypothesis is based on the grounds that in modern postindustrial societies (with delayed-return economy and developed food production and storage) frequent food-sharing may not be of a crucial importance for survival. 4. Gender differences in the proportions of egoists, egalitarianists, altruists, and mixed-strategies will be present, and boys will be less generous towards strangers (as a general gender difference in parochial predispositions). 5. The increase of number of altruists and egalitarianists with age will be observed, as a function of socialization and cultural pressure against selfishness.

## Results

**Figure 1:**
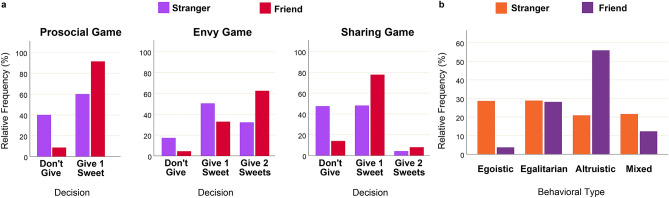
Distributions of individual decisions in the games and distributions of behavioral types towards friends and strangers. (**a**) Differences in distributions of decisions across three games are statistically significant (chi-squared test for independence: X^2^ = 4490.71, df = 4, *p* < 0.001), as well as differences in decisions towards friends and strangers within each game: Prosocial Game (chi-squared test for independence: X^2^ = 602.27, df = 1, *p* < 0.001), Envy Game (X^2^ = 485.89, df = 2, *p* < 0.001), Sharing Game (X^2^ = 614.57, df = 2, *p* < 0.001); (**b**) differences in the distributions of behavioral tendencies (Egoistic, Egalitarian, Altruistic, and Mixed) towards friends and strangers are statistically significant (chi-squared test for independence: X^2^ = 923.78, df = 3, *p* < 0.001).

The distributions of decisions in each experimental game in the general sample are presented in Fig. 1a. Generally, children and adolescents were willing to behave prosocial in the Prosocial Game: in more than 50% of the cases they gave one sweet to a partner, both under “stranger” (60%) and “friend” (91.3%) conditions, however, considerably favoring friends. The Envy Game generally delivered the least prosocial response: decision to give two sweets to a partner (with no cost for oneself) was almost as frequent (47.4%) as the decision to give one sweet (41.7%), and the deviant decisions not to give anything at all had the highest frequency in this game (10.9%) (compared to frequency of deviant prosocial decisions in Sharing (6.2%) game). However, the parochial effect in the Envy Game still remained clear and had the same direction (favoring friends) as in all other games.

The distributions of behavioral types, obtained after classification of individual decisions across all three experimental games, are presented in Fig. 1b. Generally, the frequencies of altruistic (38.4%) and egalitarian (28.5%) behavioral tendencies were higher than those of egoistic (16.1%) and mixed (17.0%) styles. However, there was a significant parochial effect, with stranger partners receiving more egoistic and mixed responses, whereas friends receiving considerably more altruistic allocations.

### Population and parochial effects

**Figure 2:**
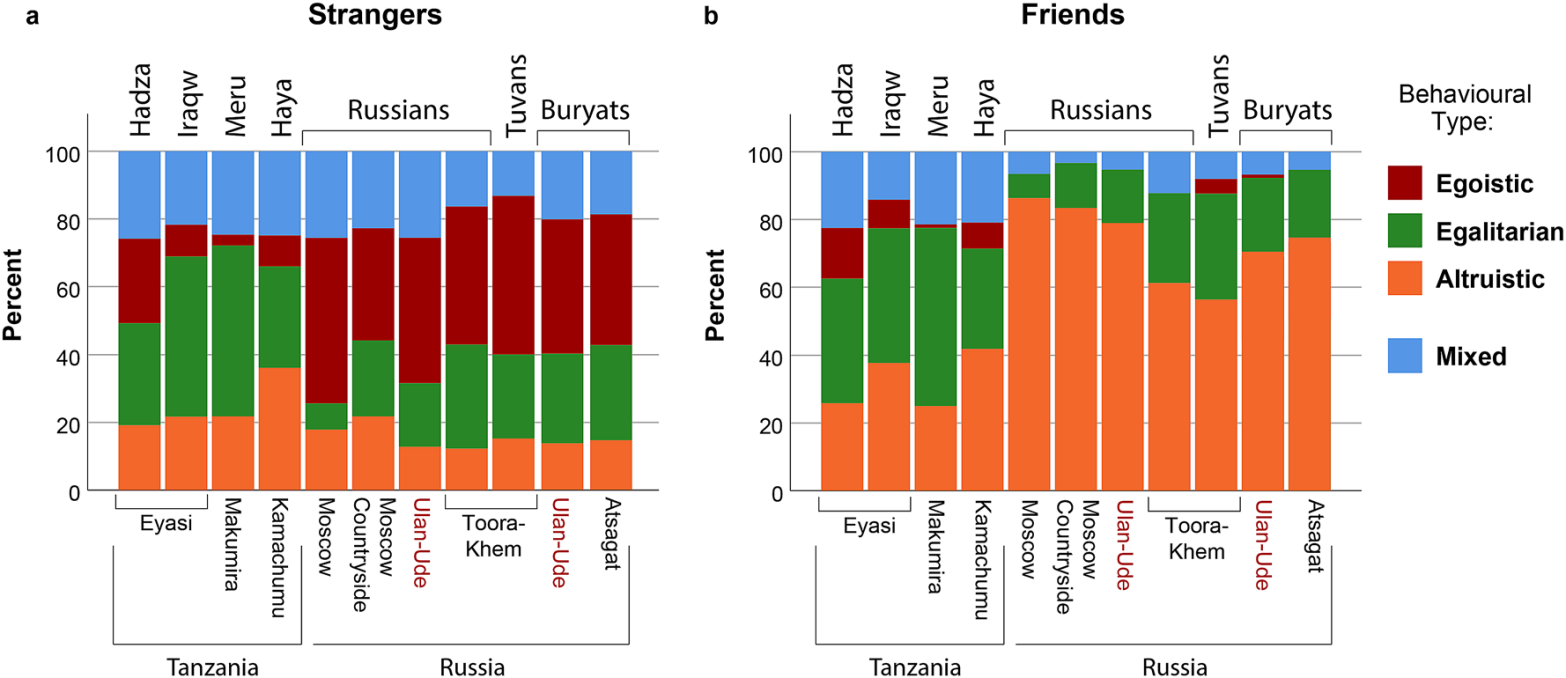
Distributions of behavioral types across ethnic groups with locations. Ethnic groups from Tanzania: Hadza, Iraqw, Meru, Haya; ethnic groups from Russia: Russians, Tuvans, Buryats. Rural locations: Eyasi, Makumira, Kamachumu, Toora-Khem, Atsagat; semi-rural location: Moscow countryside; urban locations: Moscow, Ulan-Ude. (**a**) Behavioral tendencies toward stranger partners; (**b**) behavioral tendencies toward friends.

The experimental data in our study was collected in seven economically and culturally diverse societies, including four ethnic groups from Tanzania (Hadza, Iraqw, Meru, Haya), and three ethnic groups from Russia (Russians, Buryats, Tuvans). The locations, where data were collected, differed in the levels of urbanization, and in some cases, single ethnic group could be represented by residents of both urban and rural environments, spaced apart (e.g. Russians from Moscow, Moscow countryside, Tuva, and Buryatia). In other cases, different ethnic groups could be collected in the same location (Hadza and Iraqw from Eyasi region). Due to high multicollinearity, the impacts of such factors as ethnicity, location, area (rural/urban), and country would be problematic to interpret within a single model. Therefore, first of all, our goal was to capture the most important factor(s), which could explain the observed pattern of differences in distributions of behavioral types across studied populations. Figure 2 represents the patterns of behavioral types toward stranger partners (a), and toward friends (b).

According to the observed patterns, it can be concluded that groups from Tanzania generally behaved more similar to each other, whereas groups from Russia differed from them. However, within-country variation was also observed. For instance, ethnical Russians were distinguished from other populations from the territory of Russia by complete absence of Egoistic behavioral types toward friends. To assess the relative contribution of ethnicity and location effects, we estimated the explanatory factor(s) using the Akaike information criterion (AIC). We compared three multinomial logistic regression models, where behavioral type (Egoistic, Egalitarian, Altruistic, and Mixed) was set as a response variable, and predictors (full factorial) were set as—Model 1: ethnicity by location (N = 11, see Fig. 2), and partner (stranger/friend); Model 2: general ethnicity (N = 7), area (rural/urban), and partner (stranger/friend); Model 3: general ethnicity, and partner (stranger/friend). According to the results, Model 3, which accounted only for the general ethnicity (and partner), was the best-fit model (AIC = 298.59), compared to Model 1 (AIC = 425.13), and Model 2 (AIC = 364.58). Thereafter, we used general ethnicity in the further analysis.

To assess the impacts of the ethnic group, children’s gender, age, and parochial effects on behavior in the experimental games, we ran a multinomial logistic regression, where behavioral type (Egoistic, Egalitarian, Altruistic, and Mixed) was set as a response variable, and ethnic group, children’s gender, age, and a partner type (stranger/friend) were set as predictors, along with interactions between these factors. The overall effects of the tested predictors are presented in Table 3.

**Table 3:** Effects of ethnicity, gender, age and interaction partner on behavior in the games.

Predictors	X^2^	*p *(sig.)	R^2^	*p* (model)
**Dependent variable: Behavioral Type**				
Ethnic group	488.658	< 0.001*	0.402	< 0.001
Gender	12.508	0.006		
Partner	945.139	< 0.001*		
Age	43.442	< 0.001*		
Ethnic group * Gender	58.010	< 0.001*		
Ethnic group * Partner	330.070	< 0.001*		
Ethnic group * Age	120.936	< 0.001*		
Gender * Partner	5.685	0.163		
Gender * Age	2.247	0.523		
Partner * Age	1.244	0.743		

Multinomial logistic regression: overall effects. R^2^ – Nagelkerke R^2^; statistically highly significant effects are marked with *

Since the model was very complex, more detailed parameter estimates will be presented in separate blocks, and discussed along with the visualizations, in order to facilitate understanding of the results. The full statistical details can be found in Supplementary Table 1.

Distributions of the behavioral types differed significantly between the ethnic groups. However, this was significantly interfered by parochial effect, which occurred in the general sample, and differed between specific ethnic groups (Fig. 2, Tables 3 and 4).

**Table 4:** Ethnic group and parochial effects.

Test category	Reference category	Predictors	B	Wald	*p*
**Dependent variable: behavioral type**					
Egalitarian	Egoistic	Partner (stranger)	− 2.099	12.809	< 0.001***
		Hadza * Partner (stranger)	2.227	20.284	< 0.001***
		Iraqw * Partner (stranger)	3.102	24.021	< 0.001***
		Meru * Partner (stranger)	1.811	5.039	0.025*
		Haya * Partner (stranger)	2.921	36.935	< 0.001***
		Russians * Partner (stranger)	− 17.218	3638.955	< 0.001***
Altruistic	Egoistic	Partner (stranger)	− 3.778	36.768	< 0.001
		Hadza * Partner (stranger)	3.425	41.661	< 0.001***
		Iraqw * Partner (stranger)	3.583	26.909	< 0.001***
		Meru * Partner (stranger)	2.960	12.926	< 0.001***
		Haya * Partner (stranger)	4.026	69.181	< 0.001***
		Russians * Partner (stranger)	− 17.733	2523.424	< 0.001***
		Buryats * Partner (stranger)	− 1.473	4.156	0.041*
Altruistic	Egalitarian	Partner (stranger)	− 1.679	14.068	< 0.001***
		Hadza * Partner (stranger)	1.198	8.359	0.004**
		Meru * Partner (stranger)	1.148	13.851	< 0.001***
		Haya * Partner (stranger)	1.105	15.983	< 0.001***
		Buryats * Partner (stranger)	− 0.683	4.937	0.026*

Multinomial logistic regression model: parameter estimates. Behavioral types: Egoistic, Egalitarian, Altruistic, and Mixed. Only significant results for three first categories are presented. B—regression coefficient, Wald—test statistics, *p*—statistical significance (*p < 0.05, **p < 0.01, ***p < 0.001). Full model is available in Supplementary Table 1.

Generally, egoistic and egalitarian allocations prevailed in the interactions with strangers, whereas altruistic allocations were more common in the interactions with friends. However, this tendency differed between populations. African groups demonstrated significantly higher levels of egalitarian and altruistic allocations toward strangers, compared to populations from Russia. Egoistic allocations toward strangers prevailed in Russians and Tuvans, but when interacting with friends, altruism was more pronounced in these populations (Fig. 2, Table 4). To sum up, the African ethnic groups were generally more egalitarian, whereas parochial altruism was less manifested in these populations (Hadza, Iraqw, Meru, Haya) than among Russians, Tuvans, and Buryats.

### Gender effects

The gender effects were generally weak. Males preferred the egalitarian distribution slightly more than the altruistic one, with the exception of Iraqw males, who were rather egoistic, and Meru males, who, on the contrary, were more altruistic (Table 5).

**Table 5:** Gender effects on children’s behavior.

Test category	Reference category	Predictors	B	Wald	*p*
**Dependent variable: behavioral type**					
Egalitarian	Egoistic	Iraqw * Gender (male)	− 2.215	4.174	0.041*
		Meru * Gender (male)	2.378	4.760	0.029*
Altruistic	Egoistic	Iraqw * Gender (male)	− 2.887	6.814	0.009**
		Meru * Gender (male)	3.365	9.349	0.002**
Altruistic	Egalitarian	Gender (male)	− 1.162	7.866	0.005**
		Meru * Gender (male)	0.987	10.688	< 0.001***
		Russians * Gender (male)	0.5314	3.655	0.056

Multinomial logistic regression model: parameter estimates. Behavioral types: Egoistic, Egalitarian, Altruistic, and Mixed. Only significant results for three first categories are presented. B—regression coefficient, Wald—test statistics, *p*—statistical significance (*p < 0.05, **p < 0.01, ***p < 0.001). Full model is available in Supplementary Table 1.

### Age effects

There was a significant main effect of age on the behavior of children and adolescents from the general sample, as well as a significant interaction with the ethnic group factor (Table 3). More detailed analysis revealed that age was positively associated with altruistic and egalitarian behavior and negatively with egoistic allocations. However, in most African populations this tendency was weakened, or even absent. The results of this part of the multinomial regression model are presented in Table 6 (full statistics can be found in Supplementary Table 1).

**Table 6:** Age effects on children’s behavior.

Test category	Reference category	Predictors	B	Wald	*p*
**Dependent variable: behavioral type**					
Egalitarian	Egoistic	Age	0.243	16.555	< 0.001***
		Hadza * Age	− 0.174	9.668	0.002**
		Iraqw * Age	− 0.455	9.012	0.003**
		Haya * Age	0.255	− 16.161	< 0.001***
Altruistic	Egoistic	Age	0.377	37.766	< 0.001***
		Hadza * Age	− 0.331	28.946	< 0.001***
		Iraqw * Age	− 0.829	24.197	< 0.001***
		Meru * Age	− 0.530	4.547	0.033*
		Haya * Age	− 0.430	43.196	< 0.001***
		Russians * Age	− 0.218	15.103	< 0.001***
Altruistic	Egalitarian	Age	0.134	12.230	< 0.001***
		Hadza * Age	− 0.157	9.693	0.002**
		Iraqw * Age	− 0.374	12.628	< 0.001***
		Meru * Age	− 0.245	6.799	0.009**
		Haya * Age	− 0.176	15.070	< 0.001***
		Russians * Age	− 0.171	13.791	< 0.001***

Multinomial logistic regression model: parameter estimates. Behavioral types: Egoistic, Egalitarian, Altruistic, and Mixed. Only significant results for three first categories are presented. B—regression coefficient, Wald—test statistics, *p*—statistical significance (*p < 0.05, **p < 0.01, ***p < 0.001). Full model is available in Supplementary Table 1.

**Figure 3:**
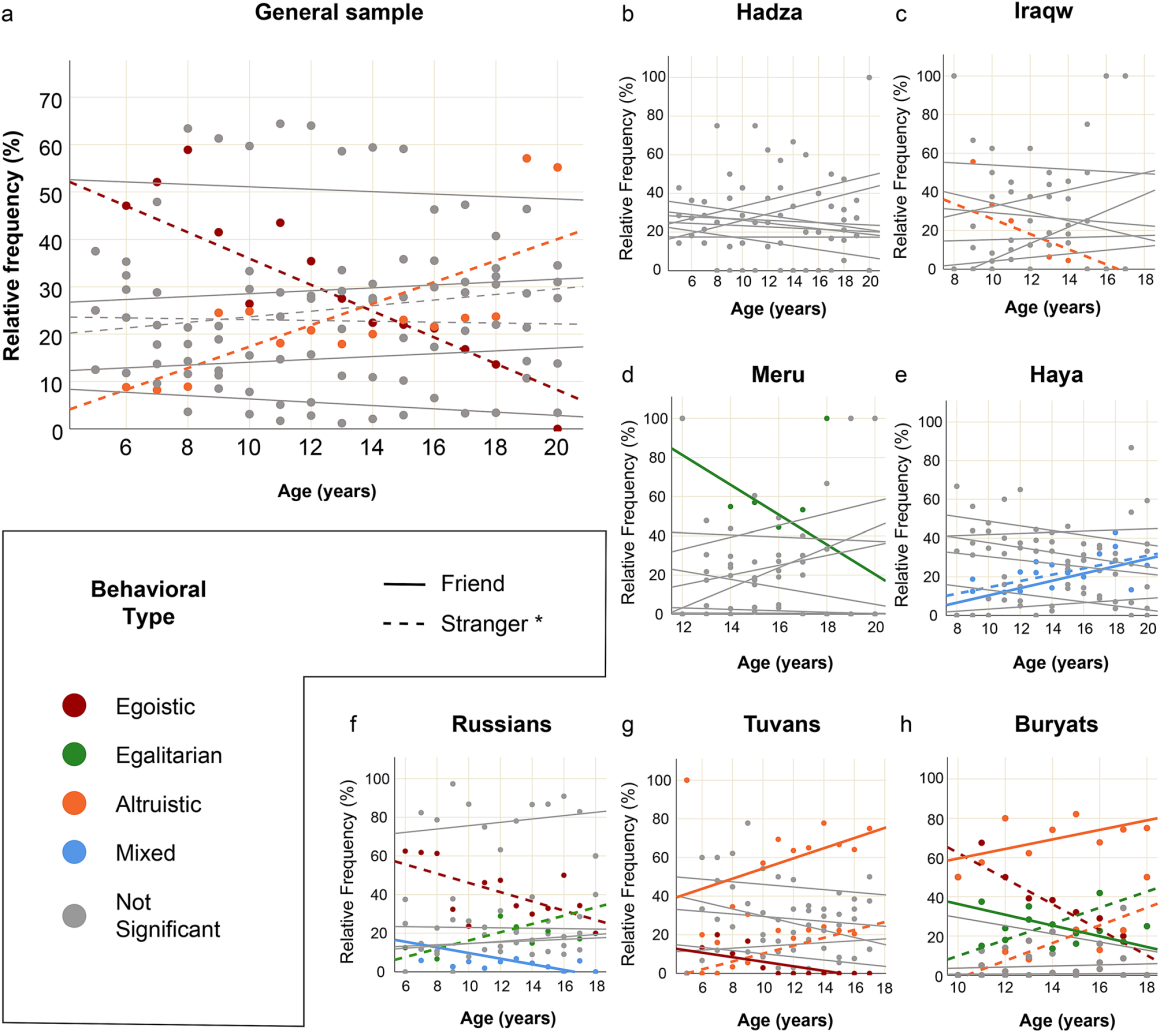
The effect of age on behavior in the games. Linear regression analysis: statistically significant associations (and strong trends) between frequencies of behavioral types and age are presented in color. * To facilitate visual perception, dashed lines for interactions with strangers are presented only for general sample and for significant associations within each ethnic group. (**a**) General sample. Egoistic [stranger]: B = − 2.773, R^2^ = 0.670, *p* < 0.001; Altruistic [stranger]: B = 2.269, R^2^ = 0.583, *p* = 0.001; associations for other behavioral types are not significant. (**b**) Hadza. No significant associations. (**c**) Iraqw. Altruistic [stranger]: B = − 3.930, R^2^ = 0.348, *p* = 0.073; associations for other behavioral types are not significant. (**d**) Meru. Egalitarian [friend]: B = − 7.615, R^2^ = 0.344, *p* = 0.097; associations for other behavioral types are not significant. (**e**) Haya. Mixed [friend]: B = 1.890, R^2^ = 0.556, *p* = 0.003; Mixed [stranger]: B = 1.636, R^2^ = 0.297, *p* = 0.054; associations for other behavioral types are not significant. (**f**) Russians. Egoistic [stranger]: B = − 2.395, R^2^ = 0.409, *p* = 0.019; Egalitarian [stranger]: B = 2.143, R^2^ = 0.352, *p* = 0.033; Mixed [friend]: B = − 1.460, R^2^ = 0.346, *p* = 0.034; associations for other behavioral types are not significant. (**g**) Tuvans. Altruistic [stranger]: B = 2.043, R^2^ = 0.670, *p* = 0.001; Egoistic [friend]: B = − 1.174, R^2^ = 0.378, *p* = 0.025; Altruistic [friend]: B = 2.629, R^2^ = 0.173, *p* = 0.157 (significance dropped due to one outlying case); associations for other behavioral types are not significant. (**h**) Buryats. Egoistic [stranger]: B = − 6.527, R^2^ = 0.848, *p* =  < 0.001; Egalitarian [stranger]: B = 4.097, R^2^ = 578, *p* = 0.017; Altruistic [stranger]: B = 4.510, R^2^ = 0.708, *p* = 0.004; Egalitarian [friend]: B = − 2.738, R^2^ = 0.402, *p* = 0.066; Altruistic [friend]: B = 2.428, R^2^ = 0.383, *p* = 0.076; associations for other behavioral types are not significant.

To assess the age effects in more detail, we ran an additional linear regression analysis, where frequency of each behavioral type toward friends and strangers per each age (in years) was set as a response variable, and age itself was set as a predictor (Fig. 3). The results for statistically significant associations are presented in the Fig. 3 legend. The full statistical details can be found in Supplementary Table 2.

According to the obtained results, there was a strong positive association between age and altruistic behavior, and strong negative association between age and egoistic behavior in the general sample, which occurred in the interactions with strangers (Fig. 3a).

Till the age of 14 for interactions with strangers, egoistic behavioral types were generally more common than altruistic, whereas above this age altruistic behavior started to overwhelm, with a wide margin for older ages. However, considering each ethnic group separately, it turned out that in African populations age was only weakly and sporadically associated with behavior: in Hadza there were no statistically significant associations at all (Fig. 3b), whereas Iraqw (Fig. 3c), Meru (Fig. 3d), and Haya (Fig. 3e) demonstrated very weak episodic associations, which could occur just by chance. At the same time, in Russians (Fig. 3f), Tuvans (Fig. 3g), and Buryats (Fig. 3h) the tendencies for decline of egoistic and increase of altruistic behavior with age were more clear and statistically significant. In Buryats egalitarian behavior toward friends decreased, whereas altruistic increased with age, however this effect was not strictly significant (statistical trend).

## Discussion

In this study we tested variations in limited resources allocation towards friends and strangers in children and adolescents from seven economically and culturally diverse societies: urban and rural societies from Russia (Russians, Buryats, Tuvans) and traditional rural societies from Tanzania (Hadza, Iraqw, Meru, and Haya). Supporting our first hypothesis, in all investigated groups all four types of individuals (egoists, egalitarianists, altruists, and mixed-strategy followers) were present. Interestingly, Russian groups were lacking individuals practicing egoistic behavior toward friends, however, altruistic allocations toward complete strangers were present in all groups with no exception. Generally, egoistic and egalitarian allocations prevailed in the interactions with strangers, whereas altruistic allocations were more common in the interactions with friends. Hence, our study supports the second (parochial) hypothesis, and confirms that children and adolescents discriminate between friends and strangers in their limited resource allocations.

Costly sharing is one of the unique manifestations of human altruism, developed as specific adaptation in modern humans^31^, and kin are the primary agents of costly cooperation^32^. However, unrelated group members and even strangers may be objects of costly sharing, although with lower probability. With the evolution towards larger social groups and general increase of communities after Neolithic revolution, natural selection may have acted positively in this direction, and the number of altruists may have been increasing. Our data on limited resource allocation in children and adolescents demonstrated that the ability for costly food sharing, exceeding the boarders of familiar social networks in all study groups.

Our results also revealed important cultural differences. African groups demonstrated significantly higher levels of egalitarian and altruistic allocations toward strangers, compared to populations from Russia. Egoistic allocations toward strangers prevailed in Russians, Tuvans, and Buryats, but when interacting with friends, altruism was more pronounced in these populations. Hence, we conclude from our data, that the African ethnic groups were generally more egalitarian, whereas parochial altruism was less manifested in these populations (Hadza, Iraqw, Meru, Haya) than in populations from the territory of Russia (Russians, Tuvans, Buryats). These results deserve special attention. Such factors as high resource stress may be possible explanations for a relatively high proportion of altruists in games with virtual strangers in African samples. Members of large-scale urban societies appeared more generous towards friends, compared to African respondents. Meantime, Africans from small-scale rural societies were more altruistic towards strangers, compared to respondents from urban societies. Hence, our third hypothesis was not confirmed. The results obtained in this study may be better understandable in light of group selection hypothesis^33,34^. In rural societies, where compatriots are living in proximity to each other in the same location and being culturally similar, there is a high probability that each unfamiliar individual they meet belong to the same ethnic (cultural) group. Recent data obtained from four pastoral ethnic groups of Kenya (Turkana, Samburu, Rendille and Borana) provided evidences that cooperation between groups may be predicted from cultural similarity^35^. Whereas, in large-scale anonymous societies, strangers may be representatives of different ethnic groups, and culturally, as well as anthropologically very distinct. Under conditions of anonymous social environment and radical shrinking of kinship network (tendency for one-child nuclear families), the value of friendship skyrocket drastically. In such case practicing costly sharing, as well as the severity of altruistic punishment for defectors^36^ may be a real necessity.

The data on gender differences in the total sample revealed a weak general effect. Males were not more parochial than females, although they preferred egalitarian rather than altruistic allocations slightly more. However, ethnic differences in this respect were obvious. E.g., the Iraqw males were more egoistic, and the Meru males were more altruistic compared to females. We suggest that gender differences in parochialism may be attributed to much extent to gender education and socialization, and cultural specificity should be noticed. Our fourth hypothesis was not confirmed by the current data.

The multinomial logistic regression model revealed a significant main effect of age on the behavior of respondents from the general sample, as well as a significant interaction with the ethnic group factor. The increase of altruistic behavior with age, as well as decrease of egoistic behavior for the whole sample were significant only in the case of interactions with strangers. Egoistic behavioral types toward strangers were more common than altruistic till 14 y., whereas above this age, altruistic behavior became prevailing over egoistic. Generally cognitive developmental differences in grasping of moral principles behind the limited resource allocation decisions may be one of explanations for these results. However, when tested for separate ethnic groups, the above-mentioned tendencies remained evident only for Russians, Tuvans and Buryats, and were manifested both in interactions with friends and strangers. For African groups these tendencies were less obvious. Hence, our fifth hypothesis was confirmed. Many studies pointed to the fact that sharing increases with age^16,24,37–40^. Morality and social norms often act against immediate individual interests, promoting interests of the society^41,42^. Norms and morality vary substantially across cultures and historically^43^, and social norms and morality principles may function as a selective force in diversifying human populations^4^. Despite obvious general similarities, ontogenetic trajectories of allocation strategies towards friends and strangers differed cross-culturally, and we suggest that adaptive learning biases may be one of possible explanations^44^. Our data pointed also to the fact that cross-cultural variations in the comprehension of positive relations between resource control and friendship aspirations during adolescence^45,46^ may be another possible explanation for current results. However, we believe that more detailed discussion in these directions is beyond the scopes of the current study.

This study has a number of limitations, which are partly due to the nature of the data collection methods. None of the samples were collected in laboratory settings, and participants were tested either in their households (as Hadza), or at school. There were two different types of schools: day schools (all samples from Russia, all primary and majority of secondary school Haya children), and boarding school children (Meru and older students from Haya sample). As we were working in some places for few days (Meru and Haya samples), it is highly probable that children who already participated in the study discussed the procedure with other peers, hence the answers obtained in later days may be biased to some extent. The effect of our presence on players’ decisions may vary as well, given that in Russia children perceive us as ordinary unfamiliar adults, while in rural Tanzania white people are rare, and children may be shyer and more hesitant. The value of suggested resources (candies) may be different for children from tested societies and these may also have affected the results obtained. We believe that this point has to be considered in more detail in future studies.

## Methods

### Participants

The study was conducted in Russia and Tanzania during the period between 2015 and 2020. In total, the data on 2,253 individuals (1,104 males and 1,149 females) within the age range of 5–18 years (M = 12,4, SD = 2,9) for samples from Russia, and of 5–20 years (M = 14,3, SD = 3,1) for samples from Tanzania was collected.

The study was conducted according to the principles expressed in the Declaration of Helsinki. The Commission for Science and Technology of Tanzania (permits: 2017-185-NA-2009-151, 2019-226-NA-2009-151, 2020-292-NA-2009-151), and the Scientific Council of the Institute of Ethnology and Anthropology of the Russian Academy of Sciences (protocol №1, dated 19 February 2015) approved the protocols used to recruit participants and data collection. All subjects and their parents provided informed consents prior to participation. Verbal consent was deemed appropriate in the case of low literacy rates in the case of Hadza and Iraqw sampling. The local school administrations were informed about the purpose of this study and also provided their consents.

### Ethnic origin of participants

The Hadza are nomadic hunter-gatherers living in northern Tanzania. They number approximately 1000–1500 individuals and live in mobile camps, each comprising an average of 30 people. Gender division of labor is obviously expressed, with men and women being the hunters and gatherers, respectively^47,48^. Although Hadza are highly fond of meat, the plant items provide more than 60% of their daily diet. The Hadza are an example of an immediate-return egalitarian society^49^. They do not produce any food items and they do not accumulate food to be consumed in the delayed future. Big-game meat, the main item widely distributed among camp members, cannot be stored by hunters for long periods because of the hot climate and absence of conservation technology^47,50^. The Hadza language contains click consonants, and classified as a language isolate^51^. The Hadza have a minimal form of religion, since they have no religious structures, leaders, ceremonies, or belief in an afterlife^47^. Sharing practices and helping behavior are common and indiscriminate^47,52^. Hadza children and adolescents in our study were living in camps together with their parents. Small children were Hadzane speakers. Most adolescents were speaking both Hadzane and Kiswahili.

The Iraqw (Wambulu) are agro-pastoralist delayed-return society who mainly live in four districts of northern Tanzania: Hanang, Babati, Mbulu and Karatu. In 1992 they were estimated about 500,000 people^53^. The Iraqw belong to a South Cushitic linguistics group of Tanzania. Most of them are Christians and visit Protestant churches more than Catholic ones, few practiced Islam. But religious syncretism is widespread among the Iraqw^54^. They have above 150 exogamous patrilineal clans^55^. Our data was collected in Endomaga boarding school, located in Mangola, Lake Eyasi region, Northern Tanzania^56^.

The Meru (Wameru, Rwa, Rwo, and Warvo) of Tanzania is a delayed-return society, one of the ethnic groups involved in intensive agriculture and now living in the south-eastern and eastern slopes of Mount Meru. Among the Meru 94 per cent of the population are Christians (75 per cent—Protestants, 25 per cent—Catholics), and 3 per cent are adherents to Islam. Anthropologically Meru is a metis population, formed by the mixing of Eastern Bantu and Maasai tribes^57^. Traditionally, Meru was patriarchal, patrilocal, and clan-organized society^57,58^. Meru children were secondary school children, living in a boarding school at the time of our data collection.

The Haya (Wahaya, Ziba, or Waziba) is the delayed-return society, one of the numerous and educated ethnic groups in Tanzania the number of which is slightly more than 2.7 million people. They are settled in the Kagera region of northwestern Tanzania on the western Lake Victoria side. The Haya language belongs to the Bantu-speaking group. The Haya are agricultural society (intensive land use) with organizing banana-based home gardens^59^. Their social organization is based on the patrilineal exogamous clans with their own animal totems and a kingship political system^60^. Along with the traditional beliefs the Haya practice Christianity, and a small number of people practice Islam^61^. Our participants were students studying at primary and secondary schools in Kagera region of Tanzania.

The Buryats is an ethnic group of Mongolian origin who were formed near Baikal Lake of Southern Siberia and mainly live in the Republic of Buryatia of Russia. The Buryats are approaching the number of above 460,000 people in Russia. They speak both Buryat and Russian languages, the former being one of the Mongolian linguistic groups. Traditionally the Buryats were a pastoral nomadic society and were divided by kinship clan groups, but nowadays most of them lead an urban lifestyle^62^. In the recent past Buryats were traditionally nomadic pastoralists with highly developed male warfare practices, and male collective hunting^63^. Buddhism is wide-spread in Buryatia (Buryatia is “the center” of Buddhism in Russia). Shamanism is also practiced by the Buryats, and some of them are Christians (Orthodox)^64^. Our data were collected in public secondary schools in rural and urban areas of Buryatia.

The Tuvans are one of the two main numerous ethnic groups in Siberia, along with the Buryats. The number of the Tuvans is estimated at about 260,000 people in Russia. They are settled in the Tuva Republic. The Tuvan language belongs to the Sayan group of the Turkic origin of the Altai family, and most Tuvans nowadays speak both Tuvan and Russian languages with Russian being especially common among the younger generation. Nomadic pastoralism persists at a substantial scale in Tuva, especially in southern regions^65^. Traditionally the Tuvans were organized in patrilineal clans, which formed primary identity of people as well as legal entitlements. After the Soviet period the patrilineal clans has become important source of social value and helping in various aspects of life (the exchanges of goods and favors between the countryside and the cities, finding a job etc.)^66^. Shamanism was the original religion of the Tuvans, and later Buddhism arrived in Tuva region^67^. Nowadays, Orthodox Christianity is also widespread among the Tuvans. The structure of the Tuvan population is not homogenous, it includes at least two major subpopulations differing both in culture, and origin. Our data was collected in the remote northeastern region of Tuva, among Tozhu Tuvans, who are the descendants of ancient pastoralists and reindeer herders. They formed as a result of the assimilation of Samoyedic and Ket groups by the Turkic-speaking populations of the region^68^. Tozhu Tuvans constitute a minority of the modern Tuvan population, and represent a Turkic subgroup with the least exposure to Mongolian influence. Our data were collected in public secondary schools in the rural area of Tuva (Toora-Khem, Todzhinsky District).

The Russians are the largest group of East Slavic ethnic groups that make up the majority of the population in Russia and are estimated above 111 million in Russia^69^. The Russian language belongs to the eastern subgroup of Slavic languages, which are part of the Indo-European language family. In the past, the main traditional occupation was arable farming. Nowadays Russians are modern industrial society. The main religious affiliation is Orthodox Christianity. Our data on Russian children and adolescents was collected in secondary public schools in Moscow region, Tuva, and Buryatia.

### Experimental design and procedure

The study design was cross-sectional. Participants provided demographic data on age, gender, ethnic origin, education, family composition, etc., and participated in a one-round session of six limited resource allocation games with virtual partners.

At the beginning of the experiment, each participant was instructed about the rules of each of the three games, so that we could make sure that a child completely understood the experiment and the consequences of different choices. All instructions were given in local native language by local assistants in order to be sure that respondents were able to comprehend the task. According to the rules, a player had to make decisions about allocation units of candies to himself/herself and/or to the partner. Every child played the Prosocial game, the Envy game and the Sharing game with imagined partners. Thus, three dilemmas were suggested: 1) Prosocial game: a player has to decide, whether to take a candy for himself, but give nothing to a potential receiver (1:0), or to take one candy for himself and give one candy to another child (1:1). In this setting, willingness to do good to another person, with no cost for oneself is tested. ‘This game serves as a measure of the basic form of prosociality, namely the willingness to avoid advantageous inequality for the benefit of the partner’^19^. As mentioned before by Fehr and his co-authors, such design suggests no costs for the decision-maker, but different motives may drive the allocation (1:1), including egalitarianism, and desire to avoid inequality, efficiency seeking, and even self-interested behavior, with the random choice of allocation decisions towards recipient^19,70,71^. 2) Envy game: a participant obtains one candy for himself, but can choose how many candies will be provided to another child: one (1:1) or two (1:2). In this setting propensity for jealousy is tested. As in the prosocial game, the decision-maker can increase the partner's payoff at no cost to himself/herself, but in the current case, such choice may result in disadvantageous inequality. 3) Sharing game: a player has to make a choice related to the real cost: he/she is suggested either to take two candies for himself/herself (2:0), or to share with other child (to give one candy) (1:1). In this case altruistic motivations of a player are tested. Contrary to previous games, the egalitarian choice here (1:1) is costly to a player, and may be interpreted as inequality aversion, thus indicating prosocial behavior. The order of the presentation of games was randomized. Each participant had been enrolled for two series of such tasks: in the first series a player had been told that the receiver was one of his/her friends (from the same class, or camp in the case of Hadza), in the second—a stranger fellow of the same age. The gender of a partner was not specified. However, respondents were told that he/she is of same age as a player. We also made it clear to the children that neither other children nor their parents/teachers would be informed about their decisions.

During the course of the first experiment (Meru children) it turned out that a considerable part of the subjects tried to violate the game rules and suggested not to share, or, alternatively, to give all candies to a partner in those cases where such options were not covered by the initial game rules. Namely, they suggested (and insisted) to give two candies to a partner in the Sharing games, which could not be prohibited, since a child could dispose of own candy at own discretion. Such decisions did not break the logics of the games and interpretation of the participants’ behavior. Another case was not giving any candies to a partner in the Envy game, without appropriation of an extra candy to oneself, which also did not break the logics of the game and could be easily interpreted. Participants provided oral explanations for the deviant decisions, which assured us that they had a clear understanding of the nature of the games, and that their choices were conscious (for example: “I would never give anything to a stranger”; “I’m not greedy, let them take all”; or “Maybe they are poorer than me, and need it more”). Since such behavior was informative, we considered it reasonable to keep these deviant decisions in the data, and further collected such decisions in all studied populations upon occurrence. Such extremely selfish or extremely prosocial deviations occurred in all studied groups with no exceptions (in total 773 deviant decisions out of total 13,518, which is 5.7%). Thus, our approach included an ethological component in that it accounted for natural predispositions of participants for certain behavior in the contexts given by the conditions of the experiment.

All subjects were then classified according to their decisions in all three games (towards friends or strangers accordingly) into four behavioral types: egoistic, egalitarian, altruistic, and mixed. The *egoistic subjects* (1) were identified as those who always preferred the allocation that minimized the partner's payoff plus maximized the positive difference in own versus partner's payoff. That is, to this category we attributed those players who were not sharing at all in all three games or gave minimum possible amount to a partner, according to the initial game rules. The *egalitarian subjects* (2) were those, preferring the egalitarian allocation (1:1) in all three games and those choosing the egalitarian allocation in all games, except the Sharing game, in which egalitarian behavior is costly (bears an element of altruism). That is, to this category belonged all players, giving one sweet in the Prosocial game, one sweet in the Envy game, and were not sharing or sharing one sweet in the Sharing game. The *altruistic subjects* (3) were those players who always selected the allocation that maximized the partner's benefits independently of the own costs (either in accordance with the game rules, or as a result of deviant decisions). That is, to this category we attributed all players who were giving one sweet in the Prosocial game, two sweets in the Envy game, and one or two sweets in the Sharing game. Those subjects whose decisions did not fit into any of the three previously mentioned behavioral types were classified as the *mixed category subjects* (4). All four behavioral types were calculated separately for the sets of plays with a potential friend or a potential stranger.

### Data analysis

General differences in distributions of individual decisions and behavioral types were assessed using a chi-squared test for independence. The impacts of independent predictors (population, gender, age, and stranger/friend type of interaction) on the behavior in the games (behavioral types) were estimated by multinomial logistic regression. Optimal predictor for assessment of the population effects was selected using Akaike information criterion. Age effects were additionally tested with linear regression models. The analysis was conducted in SPSS (Version 27).

## Supplementary Information

Supplementary Information.

## Data Availability

The dataset generated and analyzed during the current study is available in the Figshare repository, https://doi.org/10.6084/m9.figshare.20521974.
