# Supplementary material for: Variations in limited resources allocation towards friends and strangers in children and adolescents from seven economically and culturally diverse societies
Source: Sci Rep. 2022 Sep 8;12:15232. doi: 10.1038/s41598-022-19354-7 (PMC9458677; doi:10.1038/s41598-022-19354-7)
Supplement: Supplementary file 1 — Supplementary Information. [file 41598_2022_19354_MOESM1_ESM.pdf]

Variations in limited resources allocation towards friends and strangers in children and adolescents from seven economically and culturally diverse societies

Butovskaya M., Rostovtseva V., Dronova D., Burkova V., Adam Y.

Supplementary Table 1. Impact of ethnicity, gender, age and parochial effects

**Dependent variable**: Behavioral Type					
**Test** category	**Reference** category	**Predictors**	**B**	**Wald**	**p**
**Egalitarian**	Egoistic	Gender (male)	0.367	0.485	0.486
		Ethnicity			
		Hadza	0.531	0.724	0.463
		Iraqw	6.544	9.383	0.002
		Meru	4.611	1.608	0.205
		Haya	1.905	5.087	0.024
		Russians	17.539	627.206	<0.001
		Buryats	0.046	0.001	0.970
		Tuvans	0[a]		
		Partner (stranger)	-2.099	12.809	<0.001
		Age	0.243	16.555	<0.001
		Gender (male) * Age	-0.019	0.256	0.613
		Gender (male) * Partner (stranger)	-0.466	2.077	0.150
		Gender * Ethnicity			
		Gender (male) * Hadza	0.168	0.145	0.703
		Gender (male) * Iraqw	-2.215	4.174	0.041
		Gender (male) * Meru	2.378	4.760	0.029
		Gender (male) * Haya	0.302	0.590	0.442
		Gender (male) * Russians	-0.061	0.038	0.845
		Gender (male) * Buryats	-0.116	0.118	0.732
		Gender (male) * Tuvans	0[a]		
		Age * Ethnicity			
		Age * Hadza	-0.174	9.668	0.002
		Age * Iraqw	-0.455	9.012	0.003
		Age * Meru	-0.285	1.409	0.235
		Age * Haya	-0.255	16.161	<0.001
		Age * Russians	-0.047	0.781	0.377
		Age * Buryats	0.040	0.246	0.620
		Age * Tuvans	0[a]		
		Partner (stranger) * Ethnicity			
		Partner (stranger) * Hadza	2.227	20.284	<0.001
		Partner (stranger) * Iraqw	3.102	24.021	<0.001
		Partner (stranger) * Meru	1.811	5.039	0.025
		Partner (stranger) * Haya	2.921	36.935	<0.001
		Partner (stranger) * Russians	-17.218	3638.955	<0.001
		Partner (stranger) * Buryats	-0.754	1.181	0.277
		Partner (stranger) * Tuvans	0[a]		
		Partner (stranger) * Age	-0.051	1.069	0.301

Supplementary Table 1. Continued

**Dependent variable**: Behavioral Type					
**Test** category	**Reference** category	**Predictors**	**B**	**Wald**	**p**
**Altruistic**	Egoistic	Gender (male)	-0.795	2.107	0.147
		Ethnicity			
		Hadza	1.241	2.519	0.112
		Iraqw	10.578	21.231	<0.001
		Meru	5.969	2.529	0.112
		Haya	3.643	17.303	<0.001
		Russians	20.543	783.494	<0.001
		Buryats	0.728	0.331	0.565
		Tuvans	0[a]		
		Partner (stranger)	-3.778	36.768	<0.001
		Age	0.377	37.766	<0.001
		Gender (male) * Age	0.026	0.473	0.492
		Gender (male) * Partner (stranger)	-0.231	0.497	0.481
		Gender * Ethnicity			
		Gender (male) * Hadza	0.571	1.425	0.233
		Gender (male) * Iraqw	-2.887	6.814	0.009
		Gender (male) * Meru	3.365	9.349	0.002
		Gender (male) * Haya	0.677	2.939	0.086
		Gender (male) * Russians	0.453	1.968	0.161
		Gender (male) * Buryats	0.176	0.236	0.627
		Gender (male) * Tuvans	0[a]		
		Age * Ethnicity			
		Age * Hadza	-0.331	28.946	<0.001
		Age * Iraqw	-0.829	24.197	<0.001
		Age * Meru	-0.530	4.547	0.033
		Age * Haya	-0.430	43.196	<0.001
		Age * Russians	-0.218	15.103	<0.001
		Age * Buryats	0.013	0.021	0.885
		Age * Tuvans	0[a]		
		Partner (stranger) * Ethnicity			
		Partner (stranger) * Hadza	3.425	41.661	<0.001
		Partner (stranger) * Iraqw	3.583	26.909	<0.001
		Partner (stranger) * Meru	2.960	12.926	<0.001
		Partner (stranger) * Haya	4.026	69.181	<0.001
		Partner (stranger) * Russians	-17.733	2523.424	<0.001
		Partner (stranger) * Buryats	-1.473	4.156	0.041
		Partner (stranger) * Tuvans	0[a]		
		Partner (stranger) * Age	-0.030	0.358	0.550

Supplementary Table 1. Continued

**Dependent variable**: Behavioral Type					
**Test** category	**Reference** category	**Predictors**	**B**	**Wald**	**p**
**Mixed**	Egoistic	Gender (male)	-0.513	0.812	0.367
		Ethnicity			
		Hadza	0.372	0.219	0.640
		Iraqw	4.029	3.013	0.083
		Meru	2.517	0.451	0.502
		Haya	-0.190	0.040	0.842
		Russians	17.266	535.662	<0.001
		Buryats	-2.373	3.099	0.078
		Tuvans	0[a]		
		Partner (stranger)	-1.599	6.471	0.011
		Age	0.099	2.200	0.138
		Gender (male) * Age	0.006	0.022	0.881
		Gender (male) * Partner (stranger)	-0.052	0.023	0.879
		Gender * Ethnicity			
		Gender (male) * Hadza	-0.006	<0.001	0.990
		Gender (male) * Iraqw	-2.354	4.383	0.036
		Gender (male) * Meru	3.208	8.309	0.004
		Gender (male) * Haya	0.542	1.485	0.223
		Gender (male) * Russians	0.790	5.140	0.023
		Gender (male) * Buryats	0.375	0.878	0.349
		Gender (male) * Tuvans	0[a]		
		Age * Ethnicity			
		Age * Hadza	-0.069	1.197	0.274
		Age * Iraqw	-0.195	1.347	0.246
		Age * Meru	-0.103	0.174	0.677
		Age * Haya	-0.016	0.051	0.821
		Age * Russians	-0.007	0.013	0.910
		Age * Buryats	0.250	7.571	0.006
		Age * Tuvans	0[a]		
		Partner (stranger) * Ethnicity			
		Partner (stranger) * Hadza	1.690	9.684	0.002
		Partner (stranger) * Iraqw	2.443	11.801	0.001
		Partner (stranger) * Meru	1.040	1.516	0.218
		Partner (stranger) * Haya	2.162	16.498	<0.001
		Partner (stranger) * Russians	-16.902	2022.424	<0.001
		Partner (stranger) * Buryats	-0.831	1.247	0.264
		Partner (stranger) * Tuvans	0[a]		
		Partner (stranger) * Age	-0.036	0.457	0.499

Supplementary Table 1. Continued

**Dependent variable**: Behavioral Type					
**Test** category	**Reference** category	**Predictors**	**B**	**Wald**	**p**
**Altruistic**	Egalitarian	Gender (male)	-1.162	7.866	0.005
		Ethnicity			
		Hadza	0.710	1.085	0.298
		Iraqw	4.034	10.653	0.001
		Meru	1.358	0.969	0.325
		Haya	1.693	7.494	0.006
		Russians	3.004	26.102	<0.001
		Buryats	0.683	0.564	0.453
		Tuvans	0[a]		
		Partner (stranger)	-1.679	14.068	<0.001
		Age	0.134	12.230	<0.001
		Gender (male) * Age	0.021	0.467	0.494
		Gender (male) * Partner (stranger)	0.235	2.009	0.156
		Gender * Ethnicity			
		Gender (male) * Hadza	0.403	0.984	0.321
		Gender (male) * Iraqw	-0.673	2.625	0.105
		Gender (male) * Meru	0.987	10.688	0.001
		Gender (male) * Haya	0.375	1.942	0.163
		Gender (male) * Russians	0.5314	3.655	0.056
		Gender (male) * Buryats	0.060	0.044	0.833
		Gender (male) * Tuvans	0[a]		
		Age * Ethnicity			
		Age * Hadza	-0.157	9.693	0.002
		Age * Iraqw	-0.374	12.628	<0.001
		Age * Meru	-0.245	6.799	0.009
		Age * Haya	-0.176	15.070	<0.001
		Age * Russians	-0.171	13.791	<0.001
		Age * Buryats	-0.027	0.166	0.684
		Age * Tuvans	0[a]		
		Partner (stranger) * Ethnicity			
		Partner (stranger) * Hadza	1.198	8.359	0.004
		Partner (stranger) * Iraqw	0.481	1.330	0.249
		Partner (stranger) * Meru	1.148	13.851	<0.001
		Partner (stranger) * Haya	1.105	15.983	<0.001
		Partner (stranger) * Russians	-0.515	3.252	0.071
		Partner (stranger) * Buryats	-0.683	4.937	0.026
		Partner (stranger) * Tuvans	0[a]		
		Partner (stranger) * Age	0.021	0.467	0.494

Supplementary Table 1. Continued

**Dependent variable**: Behavioral Type					
**Test** category	**Reference** category	**Predictors**	**B**	**Wald**	**p**
**Mixed**	Egalitarian	Gender (male)	-0.880	3.278	0.070
		Ethnicity			
		Hadza	-0.159	0.047	0.828
		Iraqw	-2.514	3.165	0.075
		Meru	-2.095	2.231	0.135
		Haya	-2.141	7.886	0.005
		Russians	-0.273	0.141	0.707
		Buryats	-2.419	4.378	0.036
		Tuvans	0[a]		
		Partner (stranger)	0.500	1.066	0.302
		Age	-0.144	7.459	0.006
		Gender (male) * Age	0.025	0.487	0.485
		Gender (male) * Partner (stranger)	0.414	4.458	0.035
		Gender * Ethnicity			
		Gender (male) * Hadza	-0.174	0.151	0.697
		Gender (male) * Iraqw	-0.140	0.084	0.771
		Gender (male) * Meru	0.831	5.022	0.025
		Gender (male) * Haya	0.240	0.443	0.506
		Gender (male) * Russians	0.851	5.877	0.015
		Gender (male) * Buryats	0.259	0.460	0.498
		Gender (male) * Tuvans	0[a]		
		Age * Ethnicity			
		Age * Hadza	0.105	3.252	0.0771
		Age * Iraqw	0.260	5.118	0.024
		Age * Meru	0.182	3.506	0.061
		Age * Haya	0.239	17.197	<0.001
		Age * Russians	0.040	0.468	0.494
		Age * Buryats	0.210	6.138	0.013
		Age * Tuvans	0[a]		
		Partner (stranger) * Ethnicity			
		Partner (stranger) * Hadza	-0.537	1.441	0.230
		Partner (stranger) * Iraqw	-0.658	1.834	0.176
		Partner (stranger) * Meru	-0.771	4.086	0.043
		Partner (stranger) * Haya	-0.759	4.174	0.041
		Partner (stranger) * Russians	0.316	0.699	0.403
		Partner (stranger) * Buryats	-0.077	0.035	0.851
		Partner (stranger) * Tuvans	0[a]		
		Partner (stranger) * Age	0.016	0.187	0.665

Supplementary Table 1. Continued

**Dependent variable**: Behavioral Type					
**Test** category	**Reference** category	**Predictors**	**B**	**Wald**	**p**
**Altruistic**	Mixed	Gender (male)	-0.283	0.325	0.569
		Ethnicity			
		Hadza	0.869	1.244	0.265
		Iraqw	6.548	17.871	<0.001
		Meru	3.453	4.518	0.034
		Haya	3.833	25.049	<0.001
		Russians	3.277	21.252	<0.001
		Buryats	3.102	6.984	0.008
		Tuvans	0[a]		
		Partner (stranger)	-2.3179	18.173	<0.001
		Age	0.278	27.190	<0.001
		Gender (male) * Age	0.020	0.329	0.566
		Gender (male) * Partner (stranger)	-0.179	0.801	0.371
		Gender * Ethnicity			
		Gender (male) * Hadza	0.577	1.442	0.230
		Gender (male) * Iraqw	-0.533	1.016	0.313
		Gender (male) * Meru	0.156	0.157	0.692
		Gender (male) * Haya	0.135	0.141	0.708
		Gender (male) * Russians	-0.337	0.950	0.330
		Gender (male) * Buryats	-0.199	0.263	0.608
		Gender (male) * Tuvans	0[a]		
		Age * Ethnicity			
		Age * Hadza	-0.262	17.696	<0.001
		Age * Iraqw	-0.634	24.075	<0.001
		Age * Meru	-0.426	14.500	<0.001
		Age * Haya	-0.414	49.797	<0.001
		Age * Russians	-0.211	12.771	<0.001
		Age * Buryats	-0.237	7.519	0.006
		Age * Tuvans	0[a]		
		Partner (stranger) * Ethnicity			
		Partner (stranger) * Hadza	1.735	12.885	<0.001
		Partner (stranger) * Iraqw	1.139	4.603	0.032
		Partner (stranger) * Meru	1.919	20.190	<0.001
		Partner (stranger) * Haya	1.864	25.009	<0.001
		Partner (stranger) * Russians	-0.831	4.888	0.027
		Partner (stranger) * Buryats	-607	2.107	0.147
		Partner (stranger) * Tuvans	0[a]		
		Partner (stranger) * Age	0.005	0.023	0.881

Multinomial logistic regression: B – coefficient, Wald – test statistics, p – statistical significance. [a] The parameter is set to zero because it is redundant.

Supplementary Table 2. Age effects

**Predictor:** Age (years)				
**Partner**	**Response var.**	**Beta**	**R^2**	**p**
General Sample				
Stranger	Egoistic behavior	-2.773	0.670	<0.001
	Egalitarian behavior	0.591	0.110	0.209
	Altruistic behavior	2.269	0.583	0.001
	Mixed	-0.091	0.003	0.838
Friend	Egoistic behavior	-0.349	0.184	0.097
	Egalitarian behavior	0.307	0.079	0.292
	Altruistic behavior	-0.257	0.009	0.724
	Mixed	0.300	0.031	0.514
Hadza				
Stranger	Egoistic behavior	-0.250	0.063	0.350
	Egalitarian behavior	-0.228	0.052	0.396
	Altruistic behavior	-0.001	<0.001	0.996
	Mixed	0.326	0.106	0.218
Friend	Egoistic behavior	-0.349	0.121	0.186
	Egalitarian behavior	0.317	0.100	0.232
	Altruistic behavior	-0.078	0.006	0.773
	Mixed	-0.087	0.008	0.106
Iraqw				
Stranger	Egoistic behavior	0.419	0.175	0.228
	Egalitarian behavior	-0.052	0.003	0.886
	Altruistic behavior	-0.590	0.348	0.073
	Mixed	0.051	0.003	0.888
Friend	Egoistic behavior	0.317	0.101	0.372
	Egalitarian behavior	-0.108	0.012	0.767
	Altruistic behavior	0.213	0.045	0.555
	Mixed	-0.182	0.033	0.615
Meru				
Stranger	Egoistic behavior	-0.478	0.229	0.193
	Egalitarian behavior	0.266	0.071	0.490
	Altruistic behavior	-0.432	0.187	0.245
	Mixed	-0.041	0.002	0.916
Friend	Egoistic behavior	-0.050	0.003	0.898
	Egalitarian behavior	-0.587	0.344	0.097
	Altruistic behavior	0.225	0.050	0.561
	Mixed	0.453	0.205	0.220

Supplementary Table 2. Continued

**Predictor:** Age (years)				
**Partner**	**Response var.**	**Beta**	**R^2**	**p**
Haya				
Stranger	Egoistic behavior	-0.391	0.153	0.187
	Egalitarian behavior	-0.270	0.073	0.373
	Altruistic behavior	0.061	0.004	0.843
	Mixed	0.545	0.297	0.054
Friend	Egoistic behavior	0.427	0.182	0.145
	Egalitarian behavior	-0.357	0.127	0.231
	Altruistic behavior	-0.377	0.142	0.204
	Mixed	0.745	0.556	0.003
Russians				
Stranger	Egoistic behavior	-0.640	0.409	0.019
	Egalitarian behavior	0.593	0.352	0.033
	Altruistic behavior	0.151	0.023	0.622
	Mixed	-0.046	0.002	0.880
Friend	Egoistic behavior			
	Egalitarian behavior	0.208	0.043	0.496
	Altruistic behavior	0.213	0.045	0.484
	Mixed	-0.589	0.346	0.034
Tuvans				
Stranger	Egoistic behavior	-0.132	0.017	0.668
	Egalitarian behavior	-0.288	0.083	0.339
	Altruistic behavior	0.819	0.670	0.001
	Mixed	0.180	0.032	0.556
Friend	Egoistic behavior	-0.615	0.378	0.025
	Egalitarian behavior	-0.197	0.039	0.519
	Altruistic behavior	0.416	0.173	0.157
	Mixed	-0.350	0.122	0.241
Buryats				
Stranger	Egoistic behavior	-6.527	0.848	<0.001
	Egalitarian behavior	4.097	0.578	0.017
	Altruistic behavior	4.510	0.708	0.004
	Mixed	-2.080	0.160	0.286
Friend	Egoistic behavior	0.040	0.009	0.812
	Egalitarian behavior	-2.738	0.402	0.066
	Altruistic behavior	2.428	0.383	0.076
	Mixed	0.268	0.027	0.670

Linear regression models with single predictor (Age) are presented. Response variable – relative frequency of behavioral type (per age in years). Beta – standardized coefficients, p – statistical significance.
